# Translation and validation of the 12-item Oxford knee score for use in Finland

**DOI:** 10.1186/s12891-017-1405-8

**Published:** 2017-02-08

**Authors:** Aleksi Reito, Anni Järvistö, Esa Jämsen, Eerik Skyttä, Ville Remes, Heini Huhtala, Mika Niemeläinen, Antti Eskelinen

**Affiliations:** 1 Coxa Hospital for Joint Replacement, Biokatu 6b, 33520 Tampere, Finland; 2 School of Medicine, University of Tampere, Lääketieteen yksikkö (Arvo), Lääkärinkatu 1, 33520 Tampere, Finland; 3 Pihlajalinna Group, Kehräsaari B, 33200 Tampere, Finland

**Keywords:** Total knee replacement, Oxford knee score, Patient reported outcome measurement

## Abstract

**Background:**

The focus in the reporting of results after total knee replacement (TKR) has changed from surgeon/radiologist-based scores to patient-reported outcome measures (PROMs). The questionnaires used in subjective outcome are often originally published in English and need to be validated in different languages. The aim of our study was to investigate the feasibility, validity, reliability, and responsiveness of the Finnish language version of the Oxford Knee Score (OKS-S) questionnaire.

**Methods:**

The original OKS questionnaire was translated using a forward/backward protocol. The OKS-S questionnaire was sent to 225 patients who were scheduled to undergo TKR surgery. The assessment was repeated 1 year after the index operation. Half of the patients also received the RAND-36 questionnaire with the OKS-S questionnaire and the other half the Knee Injury and Osteoarthritis Outcome Score (KOOS) questionnaire. 30 patients twice received the OKS-S questionnaire preoperatively for the test-retest assessment.

**Results:**

Feasibility was acceptable with a response rate of 96% in both pre- and postoperative assessments. Correlation between OKS-S questionnaire and all domains of the KOOS questionnaire and the physical domains in the RAND-36 questionnaire was high, and confirmed both good criterion and convergent validity. Content validity was good since no ceiling or floor effect was observed. In the test-retest assessment, all but 2 patients were within the 95% limits of agreement. Responsiveness was large according to effect sizes.

**Conclusions:**

Our data suggests that the OKS-S questionnaire is suitable for the assessment of both the preoperative status and the outcome of TKR in Finnish speaking patients.

## Background

The role of patient-reported outcome measures (PROMs) in the assessment of the outcome after any orthopedic intervention has been well established. Since the indication for surgery in degenerative diseases is always relative, PROMs should be implemented in the evaluation of the outcome and effectiveness of such interventions [1, 2].

Any PROM should be culturally adaptable and in ideal situations it should be translated and validated in the desired foreign language[3]. The 12-item Oxford Knee Score (OKS) questionnaire was first introduced in 1998 [4]. The OKS questionnaire was specifically designed to measure subjective outcome after total knee replacement (TKR). Since 1998, the questionnaire has been widely used in the assessment of the outcome of knee replacement surgery and especially TKR. The OKS questionnaire has been translated and validated in 12 other languages [5–15]. No pecific culture or language related problems were described in these studies. However, only the Dutch language validation of OKS has validated in in a prospective manner (ie. before and after a TKR) similar to the original work by Dawson et al. although such setting is necessary to appropriately estimate the responsiveness and effect size of a certain questionnaire [4, 12]. We therefore thought that in addition to the need to validate the OKS in Finnish language, there is a need to confirm the previous findings concerning the performance of OKS.

The aim of our study was to investigate the feasibility, validity, reliability, and responsiveness of the Finnish language version of the Oxford Knee Score (OKS-S) questionnaire.

## Methods

### Translation and pilot study

The original (English) OKS questionnaire was independently translated into Finnish by 2 professional translators. Based on these 2 independent translations, 1 researcher drafted a Finnish language OKS questionnaire. The final phrasing of the questionnaire was negotiated by a group of authors (AJ, EJ, AE). This final Finnish language version was then translated back into English by 2 professional translators who had not participated in the first English-Finnish translation process. The 2 back-translated OKS-S questionnaires were then compared to the original English language OKS questionnaire in order to assess the validity of the Finnish translation. The comparison was carried out by 1 researcher (AJ) and a native English speaker. Minor changes were made to questions 4,5,8, and 11. These changes were subsequently discussed by the study group and the final OKS-S questionnaire was accepted, as the need for a third back translation was not deemed to be necessary.

Five patients who were visiting our outpatient clinic for preoperative evaluation for TKR surgery were randomly selected for a pilot study. Of the 5 patients, 4 were female and 1 was male. The mean age was 63 years (range 51 to 72). The mean time to complete the questionnaire was 2 min and 24 s (range 1 min 20 s to 3 min 10 s). All participants deemed the questionnaire to be straightforward and easy to complete. 2 participants had trouble clearly understanding 1 question. The other wondered whether 1 was allowed to have crane to choose option Yes, easily in the item 11. The other wondered the true meaning of kneeling in the item 7.

### Patient selection

A total of 410 patients from our outpatient clinic who were scheduled to undergo TKR surgery were asked to participate in the study. Of these, 250 agreed to do so. The only inclusion criterion was that patients had to be 18 years or older. Exclusion criteria included alcohol abuse, dementia or other neurological deficit, and/or previous replacement surgery in the affected knee. 25 patients who were scheduled to undergo bilateral TKR were also excluded. Therefore, the final study cohort comprised 225 patients. A written informed consent was obtained from all patients participating in this study. We obtained permission to perform this study from the ethics committee (Regional Ethics Committee in The Pirkanmaa Hospital District´s Science Centre, ref R12034) of the hospital district in which the study was conducted.

The patients were operated on between July 2013 and January 2015. Patient demographics are shown in Table 1.

**Table 1:** Demographic data of the patients

**Gender**	**Male**	73
	**Female**	152
**Age**	Mean (SD, range)	69 (8, 43 to 88) years
**BMI**	Mean (SD, range)	30 (18 to 46) years
**Diagnosis**	Primary OA	199
	Other	26

### Questionnaires

Prior to index surgery, all patients received the Finnish language version of the OKS (OKS-S) questionnaire. The OKS questionnaire consists of 12 items specifically related to the knee. Each item has a Likert-box response key with 5 answer options. The options are graded from 0 (worst) to 4 points (best). Hence, the total score can range from 0 points being the worst possible health state to 48 points being the best possible state.

In addition to the OKS-S questionnaire, half of the patients received a Knee Injury and Osteoarthritis Outcome Score (KOOS) questionnaire (group 1) and the other half a RAND-36 quality of life questionnaire (group 2). The KOOS questionnaire comprises 5 domains with a total of 42 questions. Each question has 5 Likert-box response keys, and each domain is scored from 0 (worst) to 100 (best). To date, KOOS has not been validated in the Finnish language.

The RAND-36 questionnaire has been validated for use in the Finnish language [16]. The questionnaire comprises 8 domains with a total of 36 questions, and each question has 2 to 5 Likert-box response keys. Each question is scored from 0 (worst) to 100 (best), and the mean of all questions results in the score for each domain, which is scored similarly. The RAND-36 questionnaire is similar to the Short Form 36 (SF-36), but there is a slight difference in the scoring for the general health and pain domains [17].

The questionnaires were sent preoperatively (1–2 weeks before the surgery) and 1 year postoperatively. Before surgery, the first 30 patients were again sent the OKS-S questionnaire after an interval of 1 week in order to assess test-retest reliability.

Only those patients who returned the questionnaires fully completed were included in the analysis. No data imputation was performed and 1 or more missing items or multiple responses resulted in the exclusion of the patient from the analyses.

### Statistics

Feasibility was defined as the percentage of patients who returned the OKS-S questionnaire correctly filled out, i.e., with no missing or duplicate responses. Validity assessment included criterion, construct (convergent and divergent), and content validity. For the purposes of the criterion validity assessment, the OKS-S questionnaire was correlated against each of the KOOS and RAND-36 domains. This was done separately for pre- and postoperative settings. In an ideal situation, criterion validity expresses the correlation of the parameter with the gold standard. However, there is no gold standard with regard to PROM prior and after TKR, and hence we selected KOOS and RAND-36 domains to be used as references. The correlation between the OKS-S questionnaire and the symptoms, pain, daily living in the KOOS questionnaire, and the physical functioning, bodily pain and role limitation due to physical problems in the RAND-36 questionnaire was used to assess convergent construct validity. A similar *a priori* hypothesis for convergent validity was used in the studies by Xie et al. [5] and Haverkamp et al. [12]. The correlation between the OKS-S questionnaire and mental health and role limitation due to emotional problems in the RAND-36 questionnaire was used to assess discriminate validity, as in the study by Xie et al. [5]. Convergent and discriminate construct validity assess the theoretical basis of the parameter, and hence we hypothesized the strongest correlation in the convergent validity assessment and the lowest correlations in the discriminate validity assessment. The correlations were calculated using the Spearman rank correlation coefficient, since the majority of the scores were skewed to the left. This was further assessed by QQ-plot for each score. Content validity expresses how well the OKS-S questionnaire covers all the symptoms experienced by patients who have undergone or are scheduled for TKR. Content validity was assessed for floor and ceiling effect. Floor effect includes the proportion of patients scoring the lowest possible (0 points), whereas ceiling effect expresses the opposite, i.e., patients scoring the maximum (48 points).

Reliability assessment included test-retest ability and internal consistency. The assessment of internal consistency included the measurement of Cronbach´s alpha. Test-retest validity was investigated with a Bland-Altman plot with 95% confidence intervals and the intraclass correlation coefficient (ICC).

Responsiveness between pre- and postoperative questionnaires was assessed. Internal responsiveness was investigated using the effect size 1 (ES1) by Cohen and effect size (ES2), i.e., standardized response mean [18]. External responsiveness was not used for 2 reasons. Firstly, we did not measure any valid reference measure. Secondly, the most suitable method for the assessment of external responsiveness would be a linear regression model as suggested by Husted et al. [18], but since the majority of the scores included in our study were not normally distributed, regression statistics were not suitable. Statistical analysis was performed with R 3.2.1 (R Foundation for Statistical Computing, Vienna, Austria).

## Results

A total of 109 patients completed the OKS-S and KOOS questionnaires preoperatively (Group 1). During the 1-year follow-up, 3 patients were lost and 2 patients died. Thus, postoperative questionnaires were obtained from 104 (95%) of the patients in this group.

In Group 2, 116 patients received both the OKS-S and the RAND-36 questionnaire preoperatively. Similarly, during the 1-year follow-up 2 patients died, 2 patients did not undergo the scheduled primary surgery, and 1 patient underwent a revision surgery. Thus, post-operative questionnaires were obtained from 111 (96%) of the patients in this group.

For the test-retest assessment, 35 patients were again sent the OKS-S questionnaire 1 week after the first preoperative measurement. Fully completed questionnaires were obtained from all these patients with the exception of 1 patient who underwent the scheduled surgery shortly after the preoperative assessment of the first OKS-S questionnaire.

### Feasibility

All 225 patients returned the preoperative OKS-S questionnaires. In 9 questionnaires, item(s) were found to be missing or duplicate responses were present. This resulted in a response rate of 96%. Postoperatively, 215 out of 218 patients (98%) returned the completed OKS-S questionnaire. Missing items were also present in 5 questionnaires, which again resulted in a total response rate of 96%.

### Validity

Correlations between the OKS and KOOS domains were stronger in the preoperative assessment compared with the postoperative assessment (Table 2). The daily living domain had the strongest correlation with the OKS-S in both a preoperative and postoperative setting, but correlation with pain was also high. Correlations between the OKS and RAND-36 domains were similarly stronger in the preoperative assessment compared with the postoperative assessment. The physical domains (physical functioning, bodily pain and role limitation due to physical problems) of the RAND-36 questionnaire had the highest correlation with the OKS domains compared with the mental or emotional domains, and thus confirmed the convergent validity. Interestingly, energy and vitality had moderate correlation with the OKS-S questionnaire preoperatively, but this correlation was also noted in the postoperative assessment (Table 2).

**Table 2:** Correlation between OKS-S with KOOS and RAND-36 domains

		Preoperative	Postoperative
KOOS	Symptoms	0.503***	0.475***
	Pain	0.709***	0.630***
	Function, daily living	0.763***	0.639***
	Function, sports and recreational activities	0.437***	0.575***
	Quality of life	0.537***	0.525***
RAND-36	Physical functioning	0.679***	0.363***
	Role limitation due to physical problems	0.300**	0.292**
	Role limitation due to emotional problems	0.170	0.152
	Social functioning	0.429***	0.306**
	Mental health	0.313**	0.159
	Energy and vitality	0.439***	0.174
	Bodily pain	0.709***	0.286**
	General health perception	0.238*	0.176

***p < .0001

**p < 0.01

*p < 0.05

In the preoperative assessment, none of the patients showed either floor or ceiling effect. In the postoperative assessment, none of the patients showed floor effect but 11 (5.2%) of the patients showed ceiling effect.

### Reliability

**Fig. 1:**
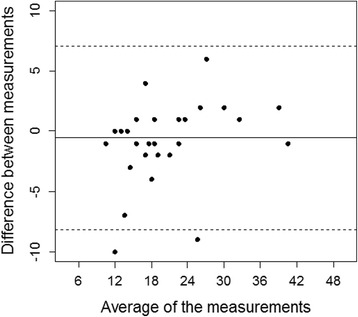
Scatter plot of the first versus the second assessment in the test-retest setting

**Fig. 2:**
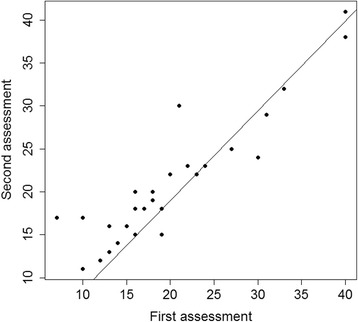
Bland-Altman plot for test-retest assessment

In the test-retest setting, the results of the first assessment correlated with the second assessment (*r* = 0.913, *p* < 0.001) (Fig. 1). A Bland-Altman plot showing the difference against the first measurement of the total scores is shown in Fig. 2. The mean of the 2 measurements was close to zero (0.57). 2 patients were out of the limits of agreement. Cronbach´s alpha was 0.85 (95% CI: 0.81 to 0.89) preoperatively and 0.90 (95% CI: 0.86 to 0.93) postoperatively. Removal of any item pre- or postoperatively did not result in any drastic change in the value of Cronbach´s alpha (range preoperative: 0.83 to 0.86, range postoperative: 0.88 to 0.90).

### Responsiveness

Median change in the OKS-S questionnaire was 18 points (range: −11 to 38). ES1 and ES2 was 2.8 and 1.1, respectively.

## Discussion

Patient-reported outcome measures (PROMs) are the most important methods used to assess the effect of treatment in orthopedic surgery. Ideally, a PROM should be reliable, practicable, and valid. The OKS questionnaire is one of the most commonly used PROMs with patients that have undergone TKR. The main advantage of the OKS questionnaire is its shortness with only 12 items in the Likert scale. To date, the OKS questionnaire has been adopted and validated in the German, Persian, Italian, Swedish, Korean, French, Japanese, Portuguese, Chinese, Singapore English, and Dutch languages (Table 3). In this study, we translated and adopted the OKS questionnaire into the Finnish language. Our results showed that the Finnish language version is a suitable tool for the assessment of both the preoperative status and the outcome of TKR.

**Table 3:** The number of patients, clinical setting and questionnaires used in the validation of OKS in non-English languages

Language	Patients	Preoperative TKR	Postoperative TKR	SF-36	VAS	EQ-5D	AKSS	WOMAC	KSS	SF-12	NHP	SIP	IKS
Italian	50	x		x									
Dutch	174	x	X	x	x		x						
Singapore English	127	x		x		x							
Chinese	131	x		x		x							
Persian	80	x		x									
Portuguese	80		X	x	x								
German	100	x						x	x	x			
Sweden	1200		X	x				x		x	x	x	
Japanese	54	x^a^		x				x					
French	100	x											x
Korean	142		X	x	x								

*VAS* Visual analogue scale, *EQ-5D* EuroQol-5D, *AKSS* American Knee Society Score, *KSS* Knee Society Score, *SF-12* Short Form 12, *NHP* Nottingham Health Profile, *SIP* Sickness Impact Profile, *IKS* International Knee Society

^a^Patients had a variety of diagnoses including OA, osteonecrosis, ligament or meniscus injury

The Short Form (36) Health Survey (SF-36) has been used in many other validation process for the assessment of convergent and discriminant construct validity (Table 3). In our study, we used the RAND-36 questionnaire, which is similar to the SF-36 except for differences in the scoring of general health and bodily pain [17]. Our results concur with earlier studies with regard to construct validity [12, 13]. The highest correlation suggesting good convergent construct validity was seen in physical functioning, bodily pain, and role limitation due to physical problems. In contrast, the lowest correlation suggesting good discriminant construct validity was seen with mental health, role limitation due to emotional problems, and general health. A direct comparison of the coefficients with other studies is not, however, possible for 2 reasons. Firstly, the study population included in the studies varied greatly. Secondly, some authors have included patients scheduled for knee replacement surgery, whereas other authors have investigated patients who had already undergone the surgery. As can be seen in our study, the results differ to some extent depending on whether the validation has been in the preoperative or postoperative setting. In addition, we observed that postoperative scores are clearly skewed to the left resulting in a small but acceptable ceiling effect. With the older scoring system (12 points best, 60 points worst) the outcome would have been skewness to the right and a small floor effect. This also results in violation of assumption of normality with the postoperative score, and thus the use of Pearson correlation coefficient is not possible. However, the Pearson correlation has been used in other studies where postoperative OKS has been correlated against SF-36 domains [12].

The OKS questionnaire has excellent criterion validity in the preoperative setting, but also has a slight ceiling effect in the postoperative setting. This is, however, well below the accepted 30%. With regard to criterion validity, we observed neither floor nor ceiling effect in the preoperative assessment. In the postoperative assessment, no floor effect was noted. However, ceiling effect was noted in 11 patients (5.2%). In the previous studies, these effects have been about the same. In accordance with our results, no ceiling or floor effect was present in the preoperative setting in Chinese, Singapore English, German, Dutch, and French language validation [5, 10, 12, 14] studies that used the same, newer scoring system (48 points for best possible outcome and 0 points for worst possible outcome), as we did. In the Swedish, Korean, and Dutch validation studies where the previous scoring system (12–60 points) was used, the percentage of patients scoring the best possible outcome tended to be slightly higher in the Dutch, Korean and Swedish language validation (9%, 1.4% and 6.8%, respectively) [12, 13, 15].

The responsiveness of the OKS-S questionnaire was large (>0.80) according to Cohen criteria [18]. In previous non-English validation studies, responsiveness has been investigated only in the Dutch language validation. In the original OKS questionnaire validation by Dawson et al. [4], the responsiveness (ES1 of 2.0) was similar to ours. A limitation of our study was that it lacked an assessment of external responsiveness. Such an assessment would, however, have required the use of a simple Likert-scale item of the patients’ global impression of change.

## Conclusions

In summary, we found that it is feasible to use the Finnish language version of the OKS questionnaire and that it has a high response rate. Furthermore, the Finnish language OKS questionnaire was also valid, and showed good convergent and divergent validity without any ceiling or floor effect. Test-retest assessment showed good reliability and responsiveness was large. In short, the psychometric performance of the OKS-S questionnaire was acceptable and our data suggests that the OKS-S questionnaire is suitable for the assessment of both the preoperative status and the outcome of TKR in Finnish speaking patients.

## Abbreviations

KOOS: Knee injury and osteoarthritis outcome score
OKS: Oxford knee score
PROM: Patient reported outcome measure
TKR: Total knee replacement

## References

[1] Marx RG (2003). Knee rating scales. Arthroscopy..

[2] Davies AP (2002). Rating systems for total knee replacement. Knee..

[3] Boynton PM. Selecting, designing, and developing your questionnaire. BMJ. 2004;328(7451):1312–1315. 10.1136/bmj.328.7451.1312 PMC420179 15166072

[4] Dawson J, Fitzpatrick R, Murray D, Carr A (1998). Questionnaire on the perceptions of patients about total knee replacement. J Bone Joint Surg Br..

[5] Xie F, Li SC, Lo NN, Yeo SJ, Yang KY, Yeo W (2007). Cross-cultural adaptation and validation of Singapore English and Chinese Versions of the Oxford Knee Score (OKS) in knee osteoarthritis patients undergoing total knee replacement. Osteoarthritis Cartilage..

[6] Takeuchi R, Sawaguchi T, Nakamura N, Ishikawa H, Saito T, Goldhahn S (2011). Cross-cultural adaptation and validation of the Oxford 12-item knee score in Japanese. Arch Orthop Trauma Surg..

[7] Ebrahimzadeh MH, Makhmalbaf H, Birjandinejad A, Soltani-Moghaddas SH (2014). Cross-cultural adaptation and validation of the persian version of the oxford knee score in patients with knee osteoarthritis. Iran J Med Sci..

[8] Goncalves RS, Tomas AM, Martins DI (2012). Cross-cultural adaptation and validation of the Portuguese version of the Oxford Knee Score (OKS). Knee..

[9] Charoencholvanich K, Pongcharoen B (2005). Oxford knee score and SF-36: translation & reliability for use with total knee arthroscopy patients in Thailand. J Med Assoc Thai..

[10] Naal FD, Impellizzeri FM, Sieverding M, Loibl M, von Knoch F, Mannion AF (2009). The 12-item Oxford Knee Score: cross-cultural adaptation into German and assessment of its psychometric properties in patients with osteoarthritis of the knee. Osteoarthritis Cartilage..

[11] Padua R, Zanoli G, Ceccarelli E, Romanini E, Bondi R, Campi A (2003). The Italian version of the Oxford 12-item Knee Questionnaire-cross-cultural adaptation and validation. Int Orthop..

[12] Haverkamp D, Breugem SJ, Sierevelt IN, Blankevoort L, van Dijk CN (2005). Translation and validation of the Dutch version of the Oxford 12-item knee questionnaire for knee arthroplasty. Acta Orthop..

[13] Dunbar MJ, Robertsson O, Ryd L, Lidgren L (2000). Translation and validation of the Oxford-12 item knee score for use in Sweden. Acta Orthop Scand..

[14] Jenny JY, Diesinger Y (2011). Validation of a French version of the Oxford knee questionnaire. Orthop Traumatol Surg Res..

[15] Eun IS, Kim OG, Kim CK, Lee HS, Lee JS (2013). Validation of the Korean version of the Oxford Knee Score in patients undergoing total knee arthroplasty. Clin Orthop Relat Res..

[16] Aalto A, Aro A, Teperi J. RAND-36 terveyteen liittyvän elämänlaadun mittarina:Mittarin luotettavuus ja suomalaiset väestöarvot. STAKES Tutkimuksia. 1999;101:1–78.

[17] Hays RD, Sherbourne CD, Mazel RM (1993). The RAND 36-Item Health Survey 1.0.. Health Econ.

[18] Husted JA, Cook RJ, Farewell VT, Gladman DD (2000). Methods for assessing responsiveness: a critical review and recommendations. J Clin Epidemiol..

